# The determinants of early childbearing by disability status in Uganda: an analysis of demographic and health survey data

**DOI:** 10.11604/pamj.2021.40.222.25348

**Published:** 2021-12-14

**Authors:** Betty Kwagala, Stephen Ojiambo Wandera

**Affiliations:** 1 Department of Population Studies, School of Statistics and Planning, College of Business and Management Sciences, Makerere University, Kampala, Uganda

**Keywords:** Early childbearing, disability status, Uganda

## Abstract

**Introduction:**

females with disabilities experience multiple sexual and reproductive challenges that can result in teenage pregnancy and motherhood. This study assessed the determinants of early childbearing among women by disability status.

**Methods:**

the study used the 2016 Uganda demographic and health survey data, analyzing a weighted sample of 18,506 women of reproductive age. We used frequency distributions to describe respondents´ characteristics, chi-squared tests and multivariable logistic regressions to establish the determinants of early childbearing.

**Results:**

early childbearing is higher among women with disabilities. The determinants of early childbearing among women with disabilities were marital status, religion, education, and occupation. The odds of early childbearing were higher among ever married compared with never married women (aOR=5.35; 95% CI: 2.42-11.84, p=0.000); women who engaged in sales and services compared with those that did not work (aOR=2.73; 95% CI: 1.36-5.50, p=0.005); and smaller religious faiths compared with protestants (aOR=2.70; 95% CI: 1.04-1.34, p=0.047). The odds reduced with advancement in education. Region, attitude towards violence and knowledge of the ovulatory cycle, though associated with early childbearing for nondisabled women were not significant for women with disabilities.

**Conclusion:**

the lack of formal education and early marriages increased the odds of early childbearing for all women. Efforts to address early childbearing especially for women with disabilities should consider advancing women´s education; and preventive measures targeting women of smaller religious faiths, stressing the dangers of early sex and marriages. The measures should target women with disabilities irrespective of attitudes towards violence, knowledge concerning fertility, and region.

## Introduction

According to the World Health Organization (WHO), about 15% of the world´s population has disabilities [1]. The prevalence of disability ranges from 4% for children under five to 17% for adults [2]. Women with disabilities experience several dimensions of marginalization based on gender, disability and sometimes wealth status [3,4], which contribute to their vulnerability to early childbearing [5-10]. WHO estimates indicate that 18.5 million girls under 19 years give birth annually in developing regions [11]. Global adolescent pregnancies are expected to increase, especially in sub-Saharan Africa by 2030 [11].

Teenage pregnancies significantly contribute to maternal and child morbidity and mortality. Up to 3.9 million adolescents in developing countries undergo unsafe abortions annually [11]. In Uganda, 25% of women begin childbearing by 19 years [2]. The prevalence of early childbearing among females with disabilities in Uganda is not known. Anecdotal sources indicate that it is high among females with hearing disabilities [12]. Persons with disabilities are often excluded from sex education, yet they may voluntarily or involuntarily engage in sexual activities which could result in conception [10,11,13]. A recent survey among women with disabilities in Uganda confirmed that 58% of adults and 36% the children had experienced sexual violence [3,14,15].

Factors associated with childbearing among young females in general include the level of education, wealth, family structure, exposure to media, alcohol and substance abuse, low self-esteem, sexual coercion, curiosity, early marriage and limited access to contraceptives [14,16-19]. Education improves access to sexual and reproductive (SRH) information and facilitates the postponement of sexual activity [16,18]. In Lao, incomplete knowledge concerning SRH was associated with early childbearing [17]. Poverty contributes to school dropout and transactional sex which can result in early childbearing [18]. Women´s disempowerment leads to risky sexual behaviors such as non-condom use [20-23].

Literature on determinants of early childbearing among women with disabilities in Uganda is scarce. Recent studies on disabilities in Uganda focused on different subjects and smaller sections of the population for instance access to HIV services by blind persons [24]; and challenges in accessing SRH services by people with physical disabilities in Kampala [25]. The study on intimate partner violence among women with disabilities considered contraceptive use, termination of pregnancy, antenatal care attendance and place of delivery [26]. This study considered early childbearing by disability status, with a special interest in women with disabilities. The analysis includes nondisabled women to establish whether there are variations in the determinants. The objective of this study is to assess the determinants of early childbearing among women by disability status.

## Methods

**Study design:** we used the 2016 Uganda Demographic and Health Survey (UDHS) data. The UDHS is based on a cross-sectional nationally representative survey design that employed a stratified two-stage cluster sampling design. The survey is representative of women age 15-49 years [2].

**Study setting:** the Uganda demographic and health survey covered the whole country, to provide the data needed to monitor and evaluate population, health, and nutrition program on a regular basis. The 2016 UDHS provides a comprehensive overview of population, maternal and child health issues [2].

**Study population:** the study included all women of reproductive in the women´s/individual recode or dataset. Men and household heads that did not fit the inclusion criteria were excluded from the analysis.

**Data and sample derivation (sample size estimation):** datasets used for this study were obtained with permission from the Demographic and Health (DHS) program website. The weighted sample was 18,506 women [2]. The study included women age 15 to 49 included in the individual recode. We merged individual (women) and peoples´ recode/datasets of the 2016 UDHS to enable us to identify women with disabilities in the individual recode. To account for the complex survey design, we used the weighting variable (v005) and the stata survey (svy) command to apply the weights to the data.

**Data collection:** collection of data that constitute the datasets used took place between June and December 2016 by Uganda Bureau of Statistics (UBOS) in partnership with the Ministry of Health, Uganda. Fertility, maternity health, including assistance at delivery were among the data collected. The data were collected using the woman and household questionnaires. The individual and people´s recodes were developed using data generated by the women and household questionnaires respectively. For further details on the data collection please see the UDHS report [2].

### Variables and measurements/definitions

**Measures of the outcome variable:** the individual recode included the age of the respondent at first birth and whether the respondent was pregnant at the time of interview. Early childbearing in this case meant conception or delivery before 18 years. Those that had delivered or were pregnant before 18 years were coded as 1 “yes” for early childbearing. The rest of the respondents were coded as 0 “no”.

**Measures of explanatory variables:** WHO defines disability as experiencing a lot of difficulty or not functioning in the domains of sight, hearing, speech, memory, walking, and personal care [27]. We adopted this definition in the generation of the variable “disability status”. Respondents were asked if they had “no difficulty”, “some difficulty”, “a lot of difficulty” or “cannot function at all” in the specified domains. There was also a provision for “don´t know” since for the household recode, women were not necessarily the respondents. These cases (9) were dropped. Disability status was recoded as “disability status" with two categories (0 no 1 yes); “no” meaning no disability and “yes” one or more disabilities. Other explanatory variables included current marital status; residence, woman´s age where the last two categories were recoded into “40+”; woman´s level of education where the last two categories were recoded into “secondary and above”; and religion where smaller groups were recoded under “other faiths”. The region was recoded into four categories because of the anticipated small numbers of women with disabilities. Some of the categories for the wealth index were merged, the poorest and poorer into one category, richer and richest also into one category to avoid having few observations in some of the original categories. Occupation was coded 1: “not working or domestic work”; 2: “professional or formal”; 3: “sales and services”; and 4: “agriculture, manual and domestic work”. Knowledge about the fertile period during the ovulatory cycle was recoded as a dichotomous variable (0 no 1 yes). Whether the respondent could refuse sex was coded as 0 “no/don´t know” 1 “yes”.

**Statistical analyses:** data were analyzed using Stata version 15. Frequency distributions were used to describe the characteristics of the respondents. We used Pearson's chi-squared (χ^2^) tests to examine the differences between the outcome and explanatory variables. For multivariable analysis, we fit binary logistic regression models for women with disabilities with nondisabled women. The level of statistical significance was set at p<0.05. Inclusion of explanatory variables for multivariable analysis was set at p=0.2. Variables to be included in the models were tested for multi-collinearity using Pearson´s correlation. Variables with issues of multi-collinearity (in this case age) were excluded from the models. We used the link test for model specification. Multivariable analysis results are presented in form of odds ratios (OR) at 95% confidence intervals, p values inclusive.

**Ethical considerations:** the informed consent form (ICF) Institutional Review Board (IRB) reviewed and approved the 2016 Uganda Demographic and Health Survey. The ORC MACRO, ICF Macro, and ICF IRBs complied with the United States Department of Health and Human Services regulations for the protection of human research subjects (45 CFR 46). Informed consent was obtained from all respondents. Participation was voluntary and anonymity was maintained by the exclusion of participants´ identifiers from the dataset [2].

## Results

**Descriptive results:** Table 1 presents the descriptive results. The majority (70%) of the respondents were below 30 years; currently or ever married (78%), Christian (over 84%), and rural residents (73%). The majority (67%) had primary or no education, and engaged in agriculture, manual or domestic work (55%). Concerning attitudes justifying spousal physical violence, 51% did not support wife beating. The majority indicated that they could refuse to have sex with a partner if they knew he had other women (76%), and had incorrect information about the ovulatory cycle (78%). Women with disabilities constituted 4% of the respondents. Early childbearing stood at 33% for women with disabilities and 29% for nondisabled women.

**Table 1:** percentage distribution of explanatory factors and early childbearing in Uganda

	All women		Early childbearing by disability status	
Explanatory variables	Frequency (n=18,506)	% of women	Women with disabilities (n=701)	Non-disabled women (n=17805)
**Age new**			**p<0.001**	**p<0.001**
15-24	8,086	43.7	19.0	21.9
25-29	3,051	16.5	42.7	31.4
30-34	2,543	13.7	36.5	35.2
35-39	2,011	10.9	43.7	38.7
40+	2,814	15.2	32.6	36.6
**Currently/formerly/never in union**			**p<0.001**	**p<0.001**
Never married	4,783	25.8	7.3	6.6
Currently or ever married	13,723	74.2	38.1	37.2
**Type of place of residence**			**P = 0.071**	**p<0.001**
Urban	4,943	26.7	26.0	22.1
Rural	13,563	73.3	34.2	31.8
**Region**			**P = 0.207**	**p<0.001**
Central	5,481	29.6	31.9	26.4
Eastern	4,879	26.4	38.1	33.7
Northern	3,546	19.2	36.4	31.4
Western	4,600	24.9	28.3	26.0
**Religion**			**P = 0.227**	**P = 0.098**
Anglican	5,774	31.2	31.3	29.2
Catholic	7,335	39.6	30.4	28.7
Muslim	2,388	12.9	34.7	31.7
Pentecostal/born-again	2,475	13.4	36.1	29.0
Other faiths	534	2.9	53.9	25.6
**Education**			**p<0.001**	**p<0.001**
No education	1,781	9.6	50.1	45.8
Primary	10,630	57.4	32.9	35.7
Secondary and above	6,095	32.9	15.3	13.6
**Wealth index**			**P = 0.200**	**p<0.001**
Poor	6,643	35.9	36.2	35.6
Middle	3,460	18.7	33.0	31.5
Rich	8,403	45.4	28.4	23.3
**Occupation**			**P = 0.007**	**p<0.001**
Not working	4,242	22.9	20.9	21.6
Professional or formal	1,520	8.2	22.1	16.1
Sales and services	2,612	14.1	38.0	28.0
Agriculture, manual or domestic work	10,131	54.8	20.9	34.8
**Attitudes supportive of intimate partner violence**			**P = 0.121**	**p<0.001**
No	9,437	51.0	29.5	26.7
Yes	9,069	49.0	35.6	31.8
**Can refuse sex if partner has other women**			**P = 0.113**	**P = 0.210**
No	4,461	24.1	27.5	28.3
Yes	14,045	75.9	34.3	29.5
**Knows ovulatory cycle**			**P = 0.657**	**P = 0.009**
No	14,462	78.2	32.2	29.8
Yes	4,044	21.9	34.6	27.2
Total		100.0	32.6	29.2

Table 1 also shows associations between early childbearing and independent factors by disability status. For women with disabilities, early childbearing was associated with age, marital status, level of education, and occupation. For nondisabled women, apart from religion and the respondent´s ability to refuse to have sex with a partner if she knows that he has other women, all explanatory factors were associated with early childbearing.

**Determinants of early pregnancy by disability status:** separate models for fit for women with disabilities and nondisabled women. Confounders such as marital status, level of education, wealth, and residence were included in the model. Variables that had a p-value of 0.2 or less were included in the models. Age was excluded on grounds of its high correlation with marital status. Marital status was retained owing to its strong association with childbearing. Table 2 presents the determinants of early child bearing. The determinants of early childbearing for women with disabilities were marital status, religion (marginally), level of education, and occupation. On average, among women with disabilities, the odds of early childbearing were higher among currently or ever married compared with never-married women (aOR=5.35; 95% CI: 2.42-11.84, p=0.000); and women of other faiths compared with Protestants (aOR=2.70; 95% CI: 1.04-1.34, p=0.047). The odds of early childbearing reduced with advancement in education and were lowest among women with secondary or higher education relative to women with on formal education (aOR=0.17; 95% CI: 0.07-0.38, p=0.000). Additionally, compared to women that were not working, the odds of early childbearing were higher among women in sales and services (aOR=2.73; 95% CI: 1.36-5.50, p=0.005).

**Table 2:** determinants of early childbearing by disability status

Explanatory factors	Women with disabilities (724 observations)			Nondisabled women (17782 observations)		
	aOR	P value	[95% Conf. Interval]	aOR	P value	[95% Conf. Interval]
**Marital status (rc never married)**						
Married or ever married	5.35	0.000	2.42-11.84	7.50	0.000	6.45-8.73
**Residence (rc urban)**						
Rural	1.19	0.504	0.71-2.00	1.09	0.175	0.96-1.24
**Region (rc Central)**						
Eastern	1.22	0.553	0.63-2.37	1.06	0.406	0.92-1.21
Northern	1.02	0.954	0.54-1.94	0.78	0.001	0.67-0.90
Western	0.74	0.310	0.41-1.33	0.68	0.000	0.59-0.80
**Religions (rc Protestants)**						
Catholic	0.96	0.853	0.62-1.48	0.94	0.182	0.85-1.03
Muslim	1.21	0.580	0.62-2.36	1.18	0.013	1.04-1.34
Pentecostal/born again	1.28	0.414	0.71-2.33	1.06	0.425	0.92-1.22
Other faiths	2.70	0.047	1.01-7.21	0.85	0.205	0.66-1.09
**Education (rc no education)**						
Primary	0.52	0.008	0.32-0.84	0.81	0.001	0.72-0.92
Secondary and above	0.17	0.000	0.07-0.38	0.27	0.000	0.23-0.32
**Wealth index (poor)**						
Middle	1.06	0.820	0.63-1.78	0.96	0.471	0.86-1.07
Rich	1.22	0.432	0.74-2.03	0.96	0.432	0.85-1.07
**Occupation (rc not working)**						
Professional or formal	1.82	0.278	0.62-5.36	0.72	0.002	0.59-0.89
Sales and services	2.73	0.005	1.36-5.50	1.01	0.918	0.86-1.19
Agriculture, manual or domestic work	1.72	0.061	0.98-3.02	1.01	0.851	0.89-1.16
**Attitudes supportive of intimate partner violence (rc not supportive)**						
	1.08	0.677	0.74-1.58	1.09	0.044	1.00-1.19
**Can refuse sex if partner has other women (rc no)**						
Yes	1.25	0.317	0.81-1.94	1.07	0.126	0.98-1.18
**Knows the ovulatory cycle (rc no)**						
yes	1.16	0.539	0.71-1.90	0.90	0.044	0.82-1.00

Rc: reference category; aOR: adjusted odds ratio

For nondisabled women, early childbearing was associated with all explanatory factors except residence, wealth index, and ability to refuse sex if the partner has other women. The directions of the results for marital status and level of education were similar to women with disabilities´. The odds of early childbearing were lower in Northern and Western regions compared with Central region (lowest in Western Uganda: aOR=0.68; 95% CI: 0.59-0.80, p=0.000); among women in professional or formal employment (aOR=0.72; 95% CI: 0.59-0.89, p=0.002); and those that knew the ovulatory cycle (aOR=0.90; 95% CI: 0.82-1.00, p=0.044). The odds of early childbearing were higher among Muslims compared with Protestants (aOR=1.18; 95% CI: 1.10 - 1.40, p=0.013); and women who with attitudes that were supportive of spousal violence (aOR=1.09; 95% CI: 1.00-1.19, p=0.044).

## Discussion

The objective of this study was to assess the determinants of early childbearing among women by disability status, with emphasis on women with disabilities. Early childbearing is higher among women with disabilities. The determinants of early childbearing for women with disabilities were marital status, religion, education and occupation. These factors were also significant for nondisabled women, although there were variations regarding religion and occupation. For women with disabilities, early childbearing was not associated with the region of residence, attitude towards intimate partner violence and knowledge of the ovulatory cycle, which factors were significant for nondisabled women.

Marital status is a strong determinant of early childbearing. Early marriage is a risk factor for early childbearing since in the Ugandan setting immediate conception after marriage is expected [28]. This was also observed in Ethiopia and Bangladesh [29,30]. By 18 years, 43% of Ugandan women are married [2]. Early childbearing could also result in early marriage as a remedy to the stigma attached to having children outside marriage and possibly to ensure the wellbeing of the young mother and child. Education and occupation are important indicators of socio-economic status that are usually associated with individuals´ wellbeing [16,18]. For both groups, the odds of early childbearing reduced with the advancement in educational attainment. Formal education therefore, has a mitigating influence on early childbearing for women irrespective of disability status. Formal education usually entails postponement of sexual activity and childbearing, since most schools in Uganda prohibit such among school-going females. Additionally, owing to literacy and relevant school-based programs, educated females are more likely to access sexual and reproductive health (SRH) information and services. These findings are in consonance with earlier findings for women in general [16,18,31,32].

Women´s occupation was a significant determinant of early childbearing for both categories of women but with variations by categories. Women with disabilities in sales and services in contrast to nondisabled women that were not working had higher odds of early childbearing. For women with disabilities, the findings imply that engagement in work outside the domestic sphere, in this case, sales and services that often involves mobility and interaction with a diversity of persons presents a higher risk for early childbearing. The informal sector is associated with low socio-economic status that increases the risk of early childbearing [33]. It is also possible that their work in sales and services is a result of early childbearing where the women had to work for child and self-sustenance. Such employment opportunities are often available in the informal sector where there is relative ease of entry and exit [16,18,34,35]. The finding concerning women with disabilities differs from non-disabled women and studies among other populations whose results revealed increased odds of early childbearing among women that were not working compared to those that were working [36,37] and a study among adolescents where occupation was not a significant determinant of early childbearing [38].

Our results show that religion is a significant determinant of early childbearing [34,39]. The increased odds of childbearing among women with disabilities of other faiths requires further research. For nondisabled women, Islam has been associated with early marriages and therefore early childbearing [39,40]. Our findings differ from Rutaremwa´s who (only adjusted for background factors) found no association between religion and childbearing [41]. Although the region of residence was a significant determinant of early childbearing for nondisabled women, it was not for women with disabilities. Cultural norms have a substantial influence on sexual and reproductive behaviors [42]. The reduced odds of early childbearing in Northern and Western Uganda for nondisabled women could be associated with the respective cultural norms and practices in the regions. For instance, some cultures in South-Western Uganda strictly prohibit premarital sex and childbearing [43]. Likewise, attitudes towards physical intimate partner violence and knowledge of the ovulatory cycle although significant for nondisabled women, were not significantly associated with early childbearing for women with disabilities. For nondisabled women, attitudes supportive of intimate partner physical violence, an indicator of disempowerment, increased the odds of early childbearing while knowledge of the ovulatory cycle, which is an indicator of understanding of conception and contraception issues, reduced the odds of early childbearing. Contrary to findings of the study of determinants of reporting sexually transmitted infections among women in Uganda, empowerment concerning knowledge and rights awareness reflected in not condoning intimate partner violence has a protective effect on early childbearing [44]. For women with disabilities, empowerment, and residence appear not to count. It appears vulnerabilities associated with disabilities contribute to early childbearing [10,11,13].

Limitations: our study has some limitations. It is not possible to establish causal relations, or the order of influence (what happens before or after) the data being cross-sectional. Examples include marital status and occupation. It is not possible to tell whether women were already in the relevant occupations before or after their first birth or pregnancy. Women´s attitudes towards teen pre-marital sexual intercourse, inability to resist sexual temptation, curiosity [14,16-18], which are important for this analysis were not included in the data set. Nevertheless, the study contributes to the body of knowledge on factors associated with early childbearing especially among women with disabilities. The study used a nationally representative sample. Therefore, the results are generalizable.

## Conclusion

Early childbearing is still a major problem especially among women with disabilities. The determinants of early childbearing for women with disabilities are marital status, women´s level of education, and occupation. Women of low social status require special attention. Efforts to address early childbearing especially for women with disabilities should consider advancing women´s education; sensitization of smaller religious faiths, stressing the dangers of early sex, marriages, childbearing and their prevention, and should target women with disabilities irrespective of attitudes towards violence and knowledge concerning fertility and region of residence.

### What is known about this topic

- Factors associated with early childbearing have been investigated in Uganda, some of the factors identified include low levels or lack of formal education, wealth, early marriages, family structure, exposure to media, low self-esteem, inability to resist sexual temptation, and sexual coercion.

### What this study adds

- This study assesses determinants of early childbearing by disability status;
- The determinants of early childbearing for women with disabilities are marital status, women´s level of education, and occupation;
- Unlike nondisabled women, region, attitudes towards violence and knowledge concerning fertility were did not predict early child bearing for women with disabilities; these had done by studies addressing women with disabilities in Uganda.
